# Ketamine-induced neuromuscular reactivity is associated with aging in female rhesus macaques

**DOI:** 10.1371/journal.pone.0236430

**Published:** 2020-09-21

**Authors:** Leif A. Havton, Natalia P. Biscola, Kari L. Christe, Ricki J. Colman

**Affiliations:** 1 Departments of Neurology and Neuroscience, Icahn School of Medicine at Mount Sinai, New York, NY, United States of America; 2 VA RR&D National Center for the Medical Consequences of Spinal Cord Injury and Neurology Service, James J. Peters Veterans Administration Medical Center, Bronx, NY, United States of America; 3 Department of Neurology, Icahn School of Medicine at Mount Sinai, New York, NY, United States of America; 4 California National Primate Research Center, UC Davis, Davis, CA, United States of America; 5 Wisconsin National Primate Research Center, UW Madison, Madison, WI, United States of America; 6 Department of Cell and Regenerative Biology, UW Madison, Madison, WI, United States of America; University of Nebraska Medical Center, UNITED STATES

## Abstract

Rhesus macaques represent an important species for translational and pre-clinical research studies across a multitude of disease and injury models, including aging. Ketamine anesthesia is used in humans and non-human primates but may be associated with adverse effects, including neuromuscular reactions. The effects of aging on ketamine adverse effects is not well characterized. Urodynamic recordings and electromyography (EMG) studies were performed in aged (>20 years old) and adult (3.9–14.9 years old) female rhesus macaques under an equal and light plane of sedation by constant rate infusion (CRI) of ketamine. A total of 4 of 41 adult subjects (9.7%) showed clinical signs of ketamine-induced abnormal neuromuscular reactivity, whereas a larger portion of 14 of 26 aged subjects showed similar ketamine-induced neuromuscular reactivity (53.8%; *P*< 0.001). The ketamine CRI rate was 19.8±0.9 mg/kg/h in adults and lower in aged subjects at 16.5±1.4 mg/kg/h (*P*<0.05). The ketamine CRI rate was negatively correlated with age (r = -0.30, *P*<0.05, n = 64). The incidence of ketamine reactivity or CRI rate was not different between aged pre-and post-menopausal females. EMG recordings during neuromuscular reactivity showed coordinated activation of multiple muscles, suggesting a central nervous system (CNS) mechanism for ketamine-associated neuromuscular reactivity. The incidence of ketamine-induced neuromuscular reactivity is age related but not affected by the estrous cycle in female rhesus macaques. A coordinated activation of multiple muscles, innervated by different peripheral nerves, suggests that ketamine-induced neuromuscular reactivity originates in the CNS.

## Introduction

Ketamine was initially introduced in clinical medicine as an anesthetic and has been used as a dissociative sedative for select emergency procedures [1]. Ketamine has also been considered for the treatment of select pain syndromes [2] and is undergoing evaluation for possible treatment use in depression [3]. In addition, ketamine is commonly used as an anesthetic for diagnostic and experimental procedures in non-human primates [4]. Ketamine has a rapid onset of action, a short half-life, and an excellent safety profile in primates [4–6]. As a result, it is used for many brief diagnostic and experimental procedures. Ketamine may be administered in combination with other agents, such as medetomidine or midazolam for short procedures in non-human primates [4, 7, 8]. The use of ketamine in combination with an alpha-2 receptor agonist or a benzodiazepine may provide sedation with increased musculoskeletal relaxation, a desired effect for some experimental protocols. However, the anesthesia provided by ketamine in combination with a second agent, with a different mechanism of action, may cause added suppression of reflex functions and potentially interfere with the interpretation of a variety of physiological studies in experimental protocols.

The use of ketamine as a single agent was demonstrated to be superior to flurane anesthesia for cystometrogram recordings in rhesus macaques, and ketamine has been suggested as a suitable agent for studies on lower urinary tract function [9]. When ketamine is administered by intravenous constant rate infusion (CRI) in rhesus macaques, a stable and light plane of sedation may be achieved with relative sparing of spinal reflexes and neuromuscular function, thereby allowing for both cystometrogram and external sphincter electromyography (EMG) studies in male and female subjects [10, 11]. However, the effects of aging on subject sensitivity to ketamine anesthesia has been largely unexplored in non-human primates, especially with regards to spinal reflex actions.

Therefore, we investigated the effects of aging on the prevalence of ketamine-associated neuromuscular reactivity and ketamine infusion rate to maintain a light plane of sedation and immobilization. The present study represents an expansion of scope of our ongoing physiologic and functional mapping studies of the lower urinary tract and pelvic floor in neurologically intact rhesus macaques and during aging [10–13]. Comprehensive urodynamic and EMG studies of the pelvic floor were analyzed to provide insights about possible underlying mechanisms for neuromuscular reactivity during ketamine exposures.

## Materials and methods

The studies were performed at the California National Primate Research Center (CNPRC), University of California at Davis, and at the Wisconsin National Primate Research Center (WNPRC), University of Wisconsin-Madison. Both institutions are accredited by the Association for Assessment and Accreditation of Laboratory Animal Care (AAALAC) International. All study protocols and procedures were approved by the UC Davis or UW Madison Institutional Animal Care and Use Committee (IACUC). The animal care was carried out in compliance with the *Guide for the Care and Use of Laboratory Animals* provided by the Institute for Laboratory Animal Research (2011). The animals were housed indoors in stainless steel cages with water provided *ad libitum*, and they were fed commercial chow supplemented with fresh fruits and nuts. Paired-housing attempts were made to encourage social interaction as tolerated. In addition, toys and portable objects were provided as environment enrichment. All animals were monitored daily by the center animal care staff with veterinarian support.

### Animals

A total of 67 female rhesus macaques *(Macaca mulatta)*, divided based on age into adult and aged groups, were included in the studies. Adult subjects were 3.9–14.9 years old (n = 41) and aged animals were 20.0–32.6 years of age (n = 26). All adult animals were within the normal reproductive age range and showed clinical signs of an active menstrual cycle with cyclical genital bleeding. The majority of the aged animals, aged 22.4 ± 0.5 years, also demonstrated an active menstrual cycle (n = 17), whereas a subset of the aged subjects, aged 29.1 ± 1.0 years, were post-menopausal (n = 9). Aged animals were determined to be postmenopausal if amenorrhea of over 12 months was documented. The date of menopause was determined from the date of last observed menses.

### Urodynamic and EMG studies

All subjects were first sedated by an intramuscular (IM) administration of ketamine (10 mg/kg). To prepare each animal for urodynamic and EMG studies, an intravenous (IV) catheter was placed followed by the placement of an endotracheal tube for airway protection. Ketamine was next administered at 10–12 mg/kg/hour IV by CRI, adjusting the dose for each subject as needed to achieve sedation and immobilization at the lowest possible rate of ketamine infusion. In a subset of animals with previously demonstrated ketamine-associated neuromuscular reactivity (n = 5), propofol was administered, initially by CRI at 20 mg/kg/hour and subsequently titrated to achieve light sedation and immobilization for urodynamic and EMG studies.

A triple-lumen 7-Fr transurethral bladder catheter (Life-Tech, Stafford, TX) was placed, and the cystometry and urethral pressure profile ports were individually attached to a TSD 104A pressure transducer and connected to an MP 150 Data Acquisition System (Biopac Systems, Goleta, CA). Pairs of wire-electrodes (1215A-F; Life-Tech) were inserted into the bilateral sides of the external urethral sphincter (EUS). Paired 22 gauge needles (Hamilton Company, Reno, NV) were placed as recording electrodes into the external anal sphincter (EAS), and surface patch electrodes were attached to the pelvic floor, immediately adjacent to the EAS, over the position of the levator ani muscles group. The EUS, EAS, and pelvic floor electrodes were connected to an MP 150 Data Acquisition System (Biopac System) for electromyography (EMG) recordings. For urodynamic studies, the bladder was first emptied. Next, the bladder was partially filled with saline, using a syringe attached to the fill port of the triple lumen catheter. The bladder pressure was continually monitored and raised by bladder filling from a baseline pressure of 0–5 cm H_2_O to a bladder pressure of about 20 cm H_2_O. A reflex bladder contraction associated with voiding was typically observed within 30–60 seconds of the bladder filling. Cystometrogram recordings documented the bladder and urethral pressure changes associated with bladder filling and the subsequent micturition reflex and voiding cycle. Concurrent multi-site EMG recordings were obtained from the external urethral and anal sphincters and pelvic floor muscles. A total of 2–3 reflex detrusor contractions were evoked in each subject. At the end of the procedure, the anesthesia administration was discontinued and the animals were allowed to recover.

### Statistical analysis

All data are presented as mean ± standard error (SE). For comparing two proportions, the N-1 Chi-squared test was performed using the MedCalc Comparison of Proportions Calculator (MedCalc Software, Ostend, Belgium). The non-parametric Mann Whitney U-Test was performed to compare data between groups using GraphPad Prism, version 6.07 (GraphPad Software, Inc, La Jolla, CA). To identify if there is a correlation between weight and ketamine CRI rate, a linear regression test was performed. A value of p<0.05 was considered to reflect a statistically significant difference between groups.

## Results

A series of 67 female rhesus macaques underwent clinical and physiologic examinations, including urodynamic studies and electromyography (EMG) of the external urethral and anal sphincters and pelvic floor muscles, under ketamine anesthesia. All animals were divided into adult (n = 41) and aged (n = 26) cohorts (**Table 1**).

**Table 1:** Demographic and reproductive history information.

Group	Sex	Number	Age (years)	Weight (kg)	Conceptions	Live births	Menopause
Adult	Female	n = 41	9.2±0.4	8.6±0.3	2.7±0.4	2.2±0.3	0/41
Aged	Female	n = 26	24.7±0.8	8.9±0.4	7.5±1.1	6.1±0.9	9/26
			*P*<0.001	ns	*P*<0.001	*P*<0.001	*P*<0.001

### Clinical assessments of neuromuscular function

All animals were examined after the induction of ketamine anesthesia to assess extremity and truncal muscle tone by passive extremity movement at proximal and distal joints. The animals were also monitored for signs of involuntary muscular activity throughout the subsequent urodynamic and EMG recordings, while they were maintained on a minimal dose of ketamine CRI to achieve light sedation and immobilization. Ketamine CRI was initially administered at 10–12 mg/kg/hour, and the infusion rate was adjusted at about 10–15% increments or decrements of the original dose to establish the lowest possible infusion rate to maintain sedation and immobilization.

**Fig 1:**
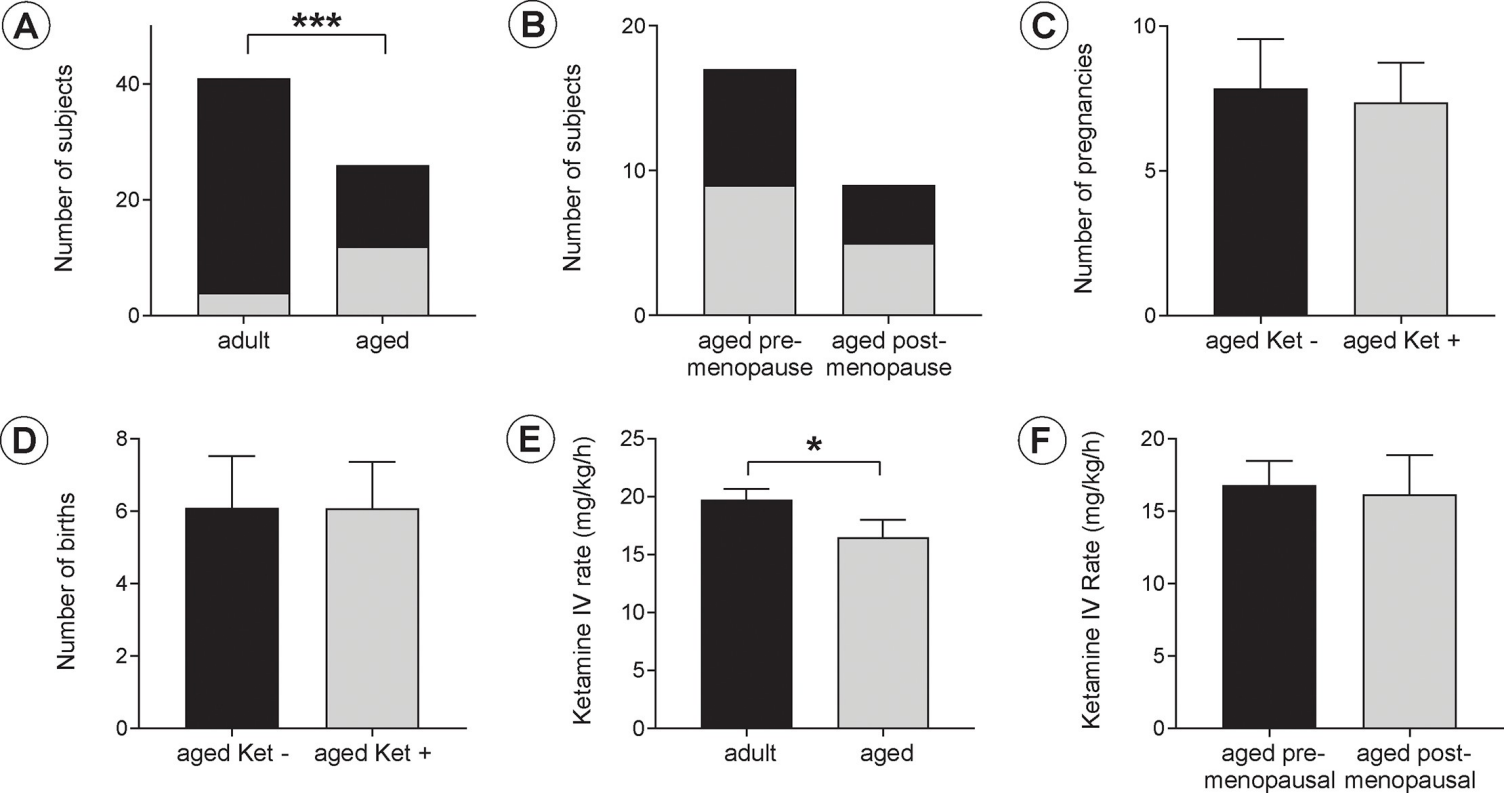
Effects of aging on ketamine-associated neuromuscular reactivity and in the infusion rate for a light plane of sedation. **A.** A total of 4/41 adult subjects (9.7%) and 14/26 aged subjects (53.8%) exhibited ketamine-associated neuromuscular reactivity and was significantly more prevalent in the aged subjects (*P*<0.001). **B.** Among the aged rhesus macaques, there was no difference in ketamine-associated neuromuscular reactivity between pre- and post-menopausal subjects. **C, D.** There was no difference in the number of pregnancies and live births for aged animals with and without ketamine-associated neuromuscular reactivity (Ket+ and Ket-, respectively. **E.** The ketamine IV infusion rate to achieve equal light planes of sedation was lower in the aged subjects (16.5±1.5 mg/kg/h) compared to the adult animals (19.8±0.9 mg/kg/h) (*P*<0.05). **F.** Among aged rhesus macaques, there was no difference in the ketamine infusion rate for equal light planes of sedation between pre- and post-menopausal subjects.

A total of 4 of the 41 adult rhesus macaques (9.7%) showed clinical signs of ketamine-induced abnormal neuromuscular activity, including an increased muscular tone and rigidity with associated joint stiffness, muscle spasms, or myoclonic activity with brief and recurrent extremity twitching movements. These abnormal neuromuscular signs were present after ketamine induction or emerged during maintenance ketamine infusion at various stages of the physiologic studies. The ketamine reaction worsened with an approximately 10–15% increase in the ketamine infusion rate and improved by a similar reduction of the rate of ketamine CRI administration. In the aged group, a significantly higher portion (14/26) of subjects showed a similar ketamine-induced abnormal neuromuscular activity (53.8%; p< 0.001) (**Fig 1A**). There was no statistical difference in the frequency of ketamine reactivity present in the aged subjects with an active menstrual cycle (9/17, 52.9%) compared to the post-menopausal animals (5/9, 55.5%) (**Fig 1B**).

With regards to potential other differences in hormonal exposures between groups, the number of pregnancies and live births were significantly higher for the aged group at 7.5±1.1 and 6.1±0.9, respectively, compared to the corresponding 2.7±0.4 pregnancies (P<0.001) and 2.2±0.3 live births (P<0.001) for the adult group (Table 1). However, in the aged group, animals with ketamine-associated neuromuscular reactivity had a history of 7.4±1.4 pregnancies and 6.1±1.3 live births (n = 14), and these reproductive considerations were not statistically different from the history of 7.7±1.6 pregnancies and 6.2±1. live births in animals without a history of similar ketamine reactivity (n = 12, ns) (**Fig 1C and 1D**).

### Ketamine infusion rate

**Fig 2:**
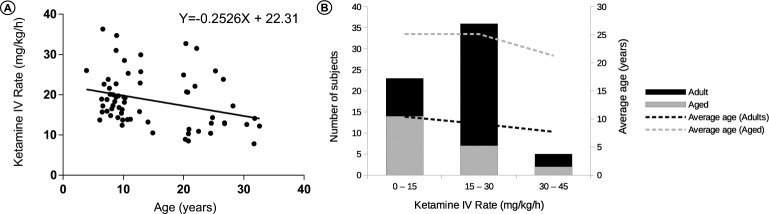
**A.** Correlation of ketamine IV infusion rate for light sedation with age. The ketamine infusion rate for achieving an equal plane of light sedation in all subjects was negatively correlated with age (r = -0.30, P<0.05, n = 64). **B.** Separation of adult and aged subjects based on low, moderate, and high rates of ketamine infusion to achieve a light plane of sedation. Ketamine was administered at 0–15 mg/kg/hr in 9 adult and 14 aged subjects, at 15–30 mg/kg/hr in 29 adult and 7 aged subjects, and at 30–45 mg/kg/hr in 3 adult and 2 aged subjects, supporting findings of a negative correlation for ketamine infusion rate with age.

The ketamine CRI administration was adjusted in each subject to provide light sedation and immobilization at the lowest possible infusion rate. The ketamine CRI rate was 19.8 ± 0.9 mg/kg/h in the adult animals (n = 41) and significantly lower in the aged subjects at 16.5 ± 1.4 mg/kg/h (n = 23; p<0.05) (**Fig 1E**). There was no statistical difference in the ketamine infusion rate between aged subjects with an active menstrual cycle and post-menopausal animals (**Fig 1F**). The ketamine infusion rate to achieve the same plane of light sedation and immobilization in all subjects was negatively correlated with age (r = -0.30, P<0.05, n = 64) (**Fig 2A**). However, several of the individual data points were located some distance from the correlation line as indicated by an R square value of 0.09. To address whether the negative correlation with age may have been influenced by uneven numbers of adult and aged subjects, all subjects were divided into groups based on ketamine infusion rates. Ketamine infusion rates were determined as low (0–15 mg/kg/hr), moderate (15–30 mg/kg/hr), or high (30–45 mg/kg/hr). Although there were more adult than aged animals in the overall sample, the vast majority of subjects with a low ketamine infusion rate consisted of aged animals (**Fig 2B**). With regards to the relationship between weight and ketamine CRI rate, a linear regression test was performed, and no significant correlation was detected.

### Urodynamic and EMG characteristics of ketamine reactivity

**Fig 3:**
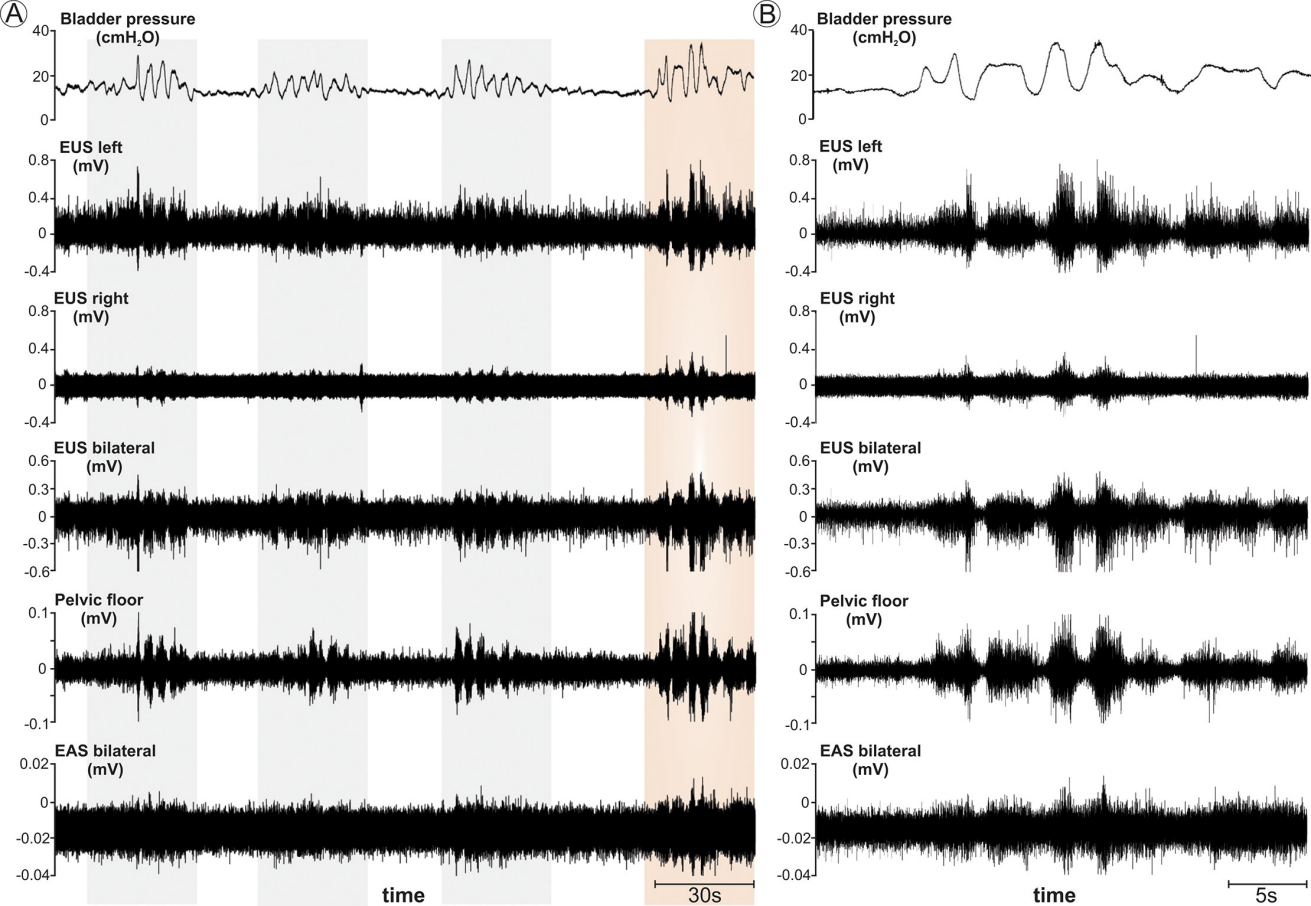
Cystometrogram and electromyography (EMG) from the external urethral and anal sphincters, and pelvic floor in a 30.5 years old rhesus macaque demonstrating generalized tonic muscular reactivity under ketamine anesthesia. **A.** Prolonged periods of neuromuscular activation with a duration of approximately 30–40 seconds are highlighted and separated by periods of relative quiescence with a duration of approximately 10–20 seconds. Note that the start and end of each period of activity is synchronized across all leads. **B.** Detail of cystometrogram and EMG recordings from period of activation in colored highlight in A. Note similar patterns of activation across all leads. EUS = external urethral sphincter; EAS = external anal sphincter.

**Fig 4:**
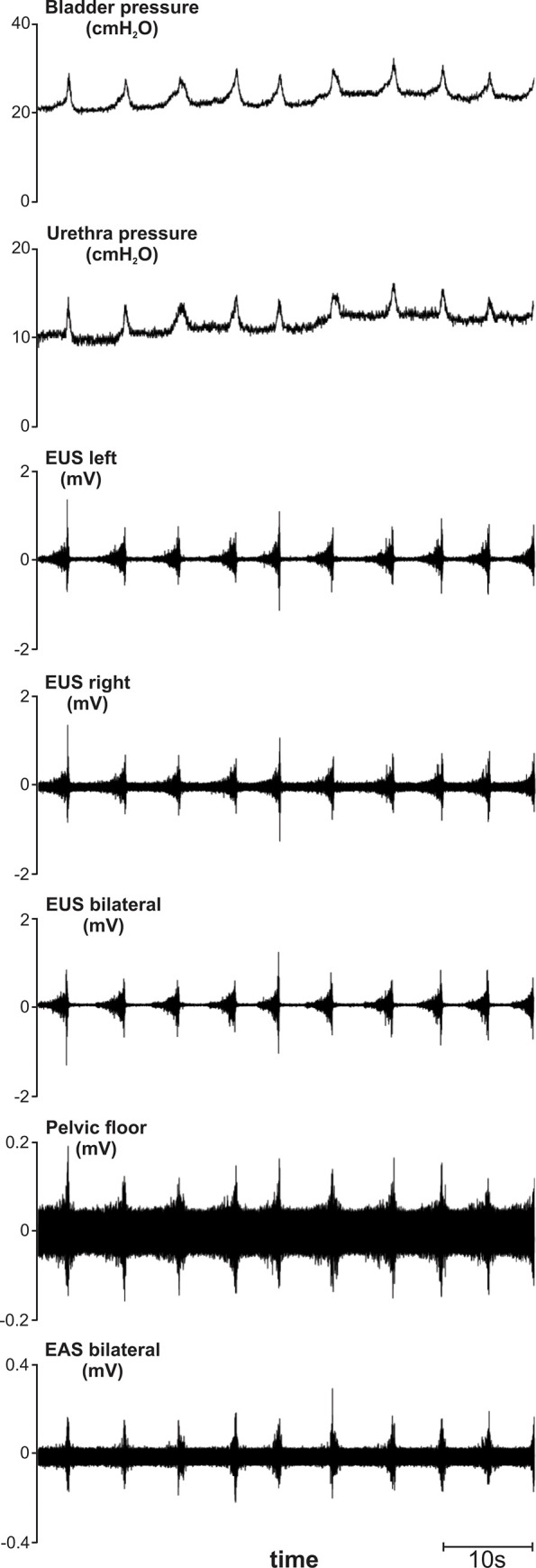
Cystometrogram, urethral pressure recordings, and electromyography (EMG) from the external urethral and anal sphincters and pelvic floor in a 24.9 years old rhesus macaque demonstrating generalized clonic muscular reactivity under ketamine anesthesia. Note synchronized brief contractions across all leads, including bladder and urethral pressure recordings and EMG recordings from the external urethral and anal sphincters and pelvic floor. EUS = external urethral sphincter; EAS = external anal sphincter.

**Fig 5:**
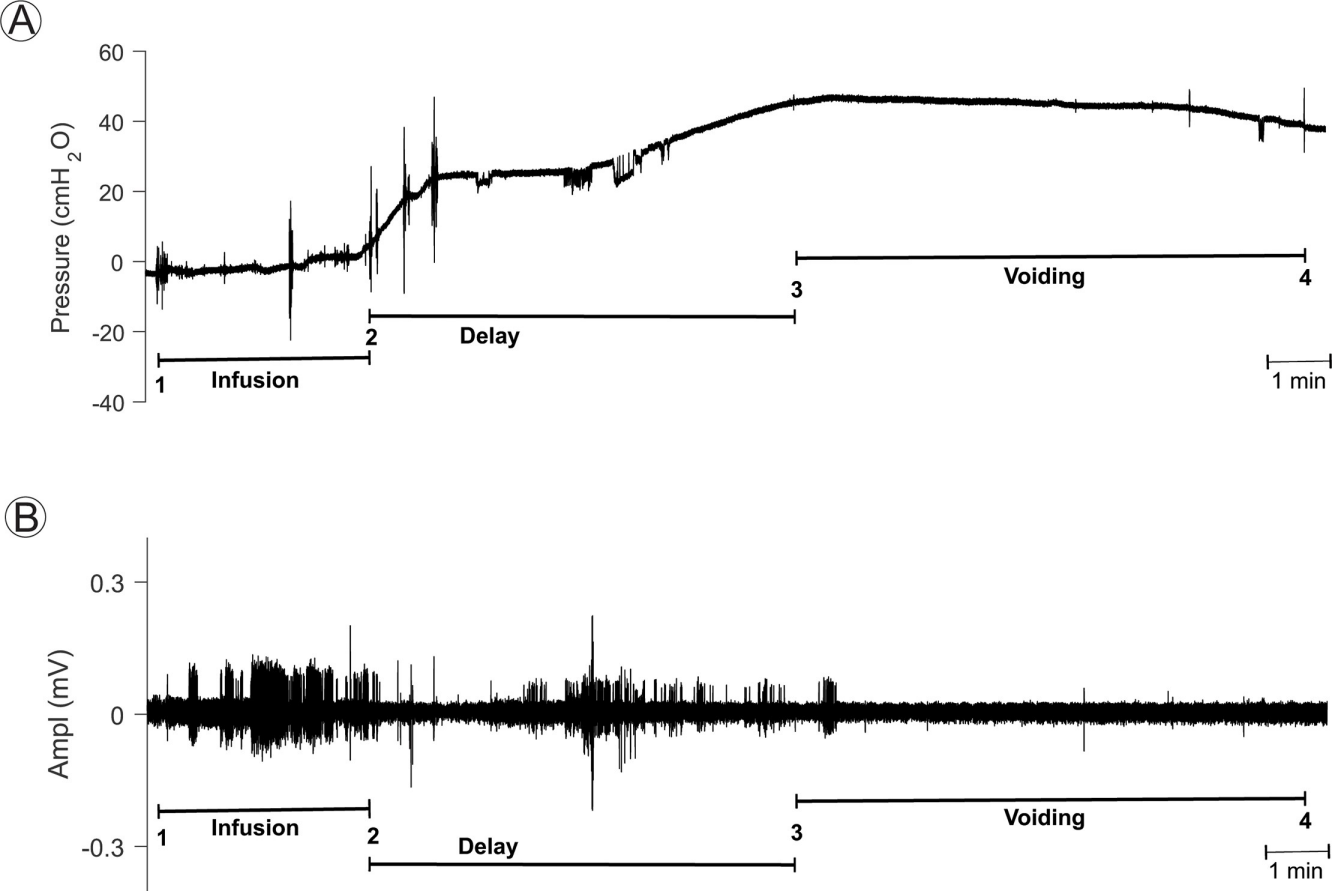
Combined cystometrogram (CMG) and external urethral sphincter (EUS) EMG recordings under propofol anesthesia in a 23.2 year old female rhesus macaque of the aged cohort. **A.** CMG recordings were performed after placement of a transurethral triple-lumen bladder catheter and emptying of bladder. Note gradual increase in bladder pressure from baseline to target of about 25 cm H_2_O in response to infusion of 145 ml saline, followed by a delay, and later onset of a reflex bladder contraction, shown by an increase in bladder pressure and subsequent voiding. Voided volume was 114 ml and voiding efficiency was 79%. **B.** EAS EMG recordings were obtained concurrently with the CMG recordings in **A**. Note EUS EMG activity during infusion of saline using the fill port of the transurethral bladder catheter. There is decreased EUS EMG activity during the early portion of the delay period, but the EUS EMG activity increased in response to the onset of the bladder contraction, consistent with the guarding reflex. The EUS EMG activity was quiescent during the voiding phase. For both **A** and **B**, onset and end of saline infusion is indicated by (1) and (2), respectively, and onset and end of voiding is indicated by (3) and (4), respectively.

All subjects underwent urodynamic studies and EMG recordings of the EUS, EAS, and levator ani muscle groups. In the subset of animals with clinical signs of ketamine-associated neuromuscular reactivity, atypical changes in bladder and urethral pressures as well as in EMG activity were demonstrated. Two patterns of atypical recordings were detected. In animals with clinical signs of generalized and fluctuating tonic muscle activation, the cystometrogram and EMG recordings were interrupted by recurrent and prolonged periods of bladder pressure changes and increased EMG activity (**Fig 3**). Each episode of tonic muscle activation and atypical electrodiagnostic activity lasted several seconds. In contrast, animals with recurrent myoclonic activity demonstrated a momentary and a brief increase in bladder pressure and burst of EMG activation, which corresponded to the observed muscular twitching (**Fig 4**). For both the tonic and clonic forms of increased neuromuscular reactivity, the onset and pause of the aberrant pressure changes and EMG activity were synchronized, as demonstrated by their simultaneous onsets and terminations across all recording sites. In a subset of animals with demonstrated ketamine reactivity (n = 5), urodynamic studies were performed under propofol anesthesia with cystometrogram and EUS EMG recordings demonstrating ability to evoke reflex micturition without neuromuscular reactivity using this alternative strategy for subject sedation (**Fig 5**).

## Discussion

The effects of age on ketamine-associated neuromuscular reactivity was determined in adult and aged female rhesus macaques undergoing urodynamic studies and EMG evaluation of the external urethral and anal sphincters and pelvic floor. The prevalence of ketamine-associated neuromuscular reactivity was significantly increased in aged compared to adult animals, but there was no difference in the prevalence of ketamine reactivity between pre- and post-menopausal animals within the aged cohort. A significantly lower infusion rate of ketamine was used in the aged compared to adult animals to establish an equal light plane of sedation, but there was no difference in the ketamine infusion rate between pre- and post-menopausal animals of the aged group. In addition, the ketamine infusion rate was negatively correlated with age. Combined urodynamic studies and EMG recordings from multiple external sphincter and pelvic floor muscles showed synchronous tonic or clonic patterns of ketamine-associated neuromuscular reactivity across all leads, suggestive of an underlying central nervous system mechanism.

Neuromuscular reactivity has been associated with ketamine sedation and its prevalence among primates may be linked to age. At higher anesthetic dosing, ketamine may result in tonic-clonic movements in over 10% of patients [14]. In a clinical study of 501 consecutive pediatric patients undergoing procedural sedation by IM administration of ketamine, a total of 6.8% of the subjects developed muscle hypertonus or clonus [15]. When ketamine was used for procedural sedation by IV administration in an emergency department cohort of 85 subjects, including both children and adults, a total of 7% of the subjects developed hypertonicity [16]. Early clinical studies of 247 cases of ketamine anesthesia for procedures showed excessive motor activity and hypertonicity in 1.9% of subjects 30 years old or younger and in 16.3% of subjects 31 years old or older [17]. The latter study suggested an age-related increase in the susceptibility for neuromuscular side effects during ketamine anesthesia. In the present study of rhesus macaques, the observed increase in the prevalence of ketamine-associated muscular hypertonicity and clonus in aged compared to adult animals supports the notion that age is a risk factor for increased neuromuscular reactivity with ketamine sedation.

The process of aging in rhesus macaques takes place about three times faster than in humans, and the animals are generally considered to be aged when they are over 20 years old [18]. In female rhesus macaques, menarche commonly takes place when the animals are about 3–5 years old, and menopause usually takes place when the animals are about 25–26 years old [19, 20]. For the present study, the adult cohort included relatively young adults, whereas the older cohort included only aged animals. Because menopause occurs at a relatively later stage in the lives of rhesus macaques compared to humans, the aged animals included both pre- and post-menopausal subjects. However, the onset of menopause did not appear to cause any additional changes in the prevalence in ketamine-associate neuromuscular reactivity or in the dose required for light sedation in the present study. Another difference between the adult and aged groups with regards to potential hormonal influences is related to reproductive history. The aged animals had a significantly larger number of pregnancies and live births compared to the adult females. The latter finding may not be surprising, as the adult animals were still in their reproductive age. However, there was no difference in the number of pregnancies and live births between the aged animals with and without ketamine-associated neuromuscular reactivity. No animal was pregnant at the time of the studies.

Ketamine exerts its principal anesthetic effect as an antagonist at N-methyl-D-aspartate (NMDA) receptors [21, 22]. Interestingly, NMDA receptor function changes in the brain during aging, and there is reduced binding to NMDA receptors at a variety of cortical and subcortical locations in aged rhesus macaques [23]. Decreased protein expression of the GluN1 subunit of the NMDA receptor has also been identified in the distal dendrites of hippocampal dentate granule cells in old rhesus macaques [24]. Both GluN1 and GluN2 proteins were shown to be decreased in neurons forming corticocortical projections in the brains of old rhesus and patas monkeys [25]. The aged-related reduction in the expression of the GluN1 subunit in the brains of elderly rhesus macaques may have implications for ketamine responses too, as the GluN1 subunit is necessary for NMDA receptor function [26]. However, additional studies are needed to determine the underlying mechanisms for the observed age-related sensitivity to ketamine.

In addition to age as a possible contributing factor influencing anesthetic and adverse effects, prior exposure to ketamine may also influence the clinical treatment response. Ketamine has been used in humans as an anesthetic agent and for treatment of pain syndromes in both adult and pediatric populations, and it is well recognized that repeat ketamine administration may result in drug tolerance [27, 28]. Repeat administration of ketamine in macaques may also result in tolerance. In early studies, a shortened period of anesthetic effect was demonstrated after the repeated administration of ketamine in rhesus macaques [29]. Tolerance was also demonstrated after administration of sub-anesthetic doses of ketamine in rhesus macaques undergoing oculomotor studies [30]. In addition, daily administration of ketamine or ketamine in combination with medetomidine resulted in physiologic and anesthetic responses consistent with the development of drug tolerance in rhesus macaques [31]. In the present study, both adults and aged subjects have had exposure to brief periods of ketamine sedation after an IM injection approximately 2–3 times per year for veterinarian health checks or occasional diagnostic or experimental procedures, but they have not received frequent or repeat ketamine dosing in close succession prior to the present studies. Furthermore, the aged animals showed an increased sensitization to ketamine, not any signs of tolerance to ketamine, as indicated by the decreased dose requirements for establishing a light plane of anesthesia.

Concurrent urodynamic recordings and EMG tracings from multiple muscle groups showed that involuntary muscle activity was rhythmic and synchronized, suggesting that the neuromuscular component of a ketamine reaction has an origin in the central nervous system (CNS) and not in the peripheral nervous tissues or at the neuromuscular junction. Prior studies have demonstrated a similarly coordinated onset of neuromuscular activity across multiple recording sites in rhesus macaques experiencing convulsive seizures associated with ketamine sedation [11, 32]. At this time, however, it remains unclear whether the presence of ketamine-associated neuromuscular reactivity may suggest an increased risk for ketamine-associated seizures in primates.

We conclude that aging is associated with an increased risk for ketamine-induced neuromuscular reactivity and that rate of ketamine infusion for a clinically equal light plane of sedation decreases with age in female rhesus macaques. There was no detectable independent effect of menopause on the ketamine-associated neuromuscular reactivity or rate of ketamine infusion to establish a light plane of sedation. Combined cystometrogram and EMG recordings showed that ketamine-associated neuromuscular reactivity was synchronized across all leads. The findings suggest that ketamine reactivity has a CNS mechanism of action but presents with a neuromuscular phenotype. Alternative forms of sedation may be considered for aged rhesus macaques or other subjects with an established or increased risk for ketamine associated neuromuscular reactivity. The present study showed that micturition reflexes can be evoked and demonstrated by cystometrogram and EUS EMG recordings under propofol anesthesia.

## Supporting information

S1 File (XLSX)
